# Exogenous Gene Expression and Insect Resistance in Dual Bt Toxin *Populus* × *euramericana* ‘Neva’ Transgenic Plants

**DOI:** 10.3389/fpls.2021.660226

**Published:** 2021-05-28

**Authors:** Yachao Ren, Xinglu Zhou, Yan Dong, Jun Zhang, Jinmao Wang, Minsheng Yang

**Affiliations:** ^1^Forest Department, Forestry College, Hebei Agricultural University, Baoding, China; ^2^Hebei Key Laboratory for Tree Genetic Resources and Forest Protection, Baoding, China

**Keywords:** Bt toxin gene, *Populus* × *euramericana* ‘Neva’, insect resistance, *Anoplophora glabripennis*, RNA-seq, differentially expressed genes

## Abstract

*Bacillus thuringiensis* (Bt) insecticidal protein genes are important tools in efforts to develop insect resistance in poplar. In this study, the *Cry1Ac* and *Cry3A* Bt toxin genes were simultaneously transformed into the poplar variety *Populus* × *euramericana* ‘Neva’ by *Agrobacterium*-mediated transformation to explore the exogenous gene expression and insect resistance, and to examine the effects of Bt toxin on the growth and development of *Anoplophora glabripennis* larvae after feeding on the transgenic plant. Integration and expression of the transgenes were determined by molecular analyses and the insect resistance of transgenic lines was evaluated in feeding experiments. Sixteen transgenic dual Bt toxin genes *Populus* × *euramericana* ‘Neva’ lines were obtained. The dual Bt toxin genes were expressed at both the transcriptional and translational levels; however, Cry3A protein levels were much higher than those of Cry1Ac. Some of the transgenic lines exhibited high resistance to the first instar larvae of *Hyphantria cunea* and *Micromelalopha troglodyta*, and the first and second instar larvae and adults of *Plagiodera versicolora*. Six transgenic lines inhibited the growth and development of *A*. *glabripennis* larvae. The differences in the transcriptomes of *A*. *glabripennis* larvae fed transgenic lines or non-transgenic control by RNA-seq analyses were determined to reveal the mechanism by which Bt toxin regulates the growth and development of longicorn beetle larvae. The expression of genes related to Bt prototoxin activation, digestive enzymes, binding receptors, and detoxification and protective enzymes showed significant changes in *A*. *glabripennis* larvae fed Bt toxin, indicating that the larvae responded by regulating the expression of genes related to their growth and development. This study lay a theoretical foundation for developing resistance to *A*. *glabripennis* in poplar, and provide a foundation for exploring the mechanism of Bt toxin action on Cerambycidae insects.

## Introduction

Poplar is an economically important tree species, but its large-scale intensive monoculture has been accompanied by increasingly serious problems of disease and insect pests. Poplar pests such as *Hyphantria cunea* (Lepidoptera), *Micromelalopha troglodyta* (Lepidoptera), and longicorn beetles can spread widely and cause substantial losses to forestry production. To reduce the losses caused by insect pests and reduce the use of chemical pesticides, the development of new insect-resistant poplar varieties using transgenic technology is particularly important (Ding et al., 2017). *Bacillus thuringiensis* (Bt) insecticidal crystal protein genes have been transformed into and expressed in many plant species and are the most widely used insect resistance genes (Lu et al., 2010; Mi et al., 2015; Yang et al., 2016). Among them, the *CryI* and *CryIII* genes, which confer high specific resistance to Lepidopteran and Coleopteran pests, respectively, are particularly prominent (Wang et al., 2018). Many studies have shown that introduction of a Bt toxin gene into transgenic poplar provides a degree of resistance to target pests (Dong et al., 2014; Xu et al., 2019, 2020); however, the introduction of a single insect resistance gene has limitations, including a narrow insecticidal spectrum and development of tolerance in the pests. To expand the insecticidal spectrum, delay the development of tolerance in pests, and improve the insect resistance of transgenic poplar, recent research has focused on the introduction of bivalent or multivalent insect resistance transgenes (Zhang B. et al., 2011; Zhang et al., 2019; Yang et al., 2016; Zhou et al., 2020).

The application of transgenic Bt toxin research to the development of insect resistance traits has made understanding the mechanism of Bt toxin action an important area of research. Bt prototoxins reach the midgut of insects and are proteolytically activated by specific proteases, releasing active toxin polypeptides which then bind to specific receptors on epithelial cells. Mechanisms proposed to be responsible for Cry cytotoxic effects include breakdown of the gut epithelium through a pore-formation mechanism in the target membrane or an alternative cell death process involving the adenylyl cyclase/PKA signaling pathway (Zhang et al., 2006; Bravo et al., 2007). These processes involve interactions with specific proteins found in the insect digestive system, including serine proteases (trypsin, chymotrypsin, and so forth) and Bt toxin-binding receptors (cadherins, aminopeptidase N, alkaline phosphatases, ABC transporter, and heat shock protein HSP70) required for pore formation (Xu et al., 2013; Jin et al., 2019). Cry-binding receptors have specific affinities for different Cry toxins (Flores-Escobar et al., 2013). In recent years, there have been many reports on the mechanism of CryI toxin (Wang et al., 2019; Shabbir et al., 2019; Fritz et al., 2020; Prabu et al., 2020), while reports on CryIII toxin are relatively fewer in number (Ruiz-Arroyo et al., 2017; Niu et al., 2020). Cry3A toxin has been shown to be toxic to Chrysomelidae insects with high mortality (Wang et al., 2012), but has only an inhibitory effect on Cerambycidae larvae with low mortality (Niu et al., 2011). The inhibitory mechanism of Cry3A toxin remains unclear and there have been few reports on the topic. Studies on transcriptome responses of insects to xenobiotics may contribute to the discovery of novel insect molecular mechanisms for detoxification and tolerance of toxins.

*Populus* × *euramericana* ‘Neva’ , a hybrid of *Populus deltoides* and *Populus nigra* L., has good material quality, fast growth, high yield, and is resistant to drought. It is a preferred poplar variety for industrial raw materials and shelter forest in China. To reduce the losses caused by insect pests and to explore the mechanism by which Bt toxin affects the growth and development of longicorn beetle larvae, two Bt toxin genes, *Cry1Ac* and *Cry3A*, were simultaneously transformed into *Populus* × *euramericana* ‘Neva’ using an *Agrobacterium*-mediated method to obtain transgenic poplar with high resistance to Lepidopteran and Coleopteran pests. Molecular analyses were performed to identify the integration and expression of exogenous genes in the transgenic lines and assessed their effects on resistance to target insects in indoor feeding experiments. Furthermore, differences in the growth of newly hatched *Anoplophora glabripennis* larvae fed with transgenic dual Bt toxin *Populus* × *euramericana* ‘Neva’ or non-transgenic *Populus* × *euramericana* ‘Neva’ were observed. The transcriptional response of *A*. *glabripennis* larvae to Bt toxin by determining transcriptional differences among larvae in different treatment groups using RNA-seq technology was also explored. These results will lay a theoretical foundation for establishing resistance to *A*. *glabripennis* larvae in poplar, and provide a basis for understanding the molecular mechanisms by which Bt toxin regulates longicorn beetle larval growth and development.

## Materials and Methods

### Plant Materials and Plant Expression Vector

**FIGURE 1:**
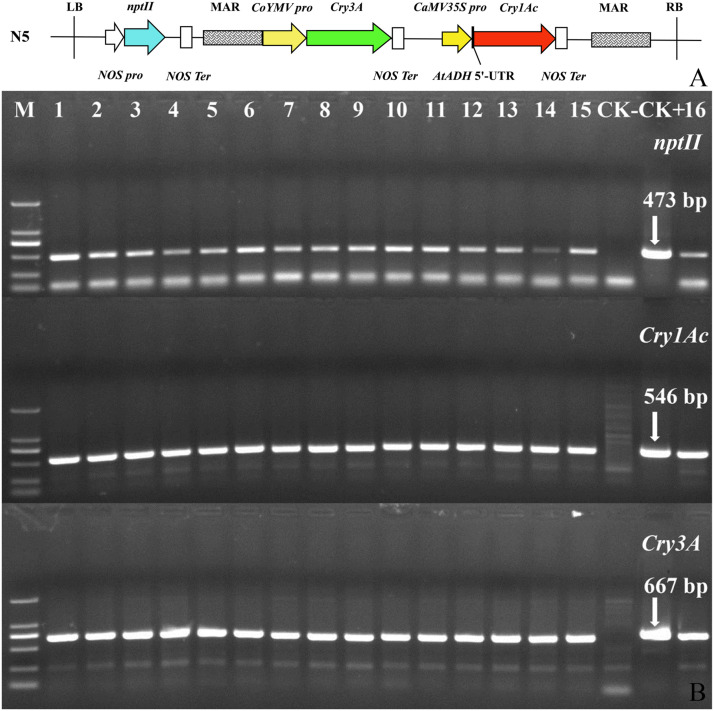
T-DNA structure of plant expression vector N5 and PCR confirmation of transgenic dual Bt toxin *Populus × euramericana* ‘Neva’ lines. **(A)** T-DNA structure of plant expression vector N5. Each target gene has a respective promoter and termination sequence in the vector, and the genes are located between the left and right T-DNA borders in the vector. N5, the name of the plant expression vector; LB, vector left boundary; *NOS pro*, promoter of neomycin phosphotransferase gene; *nptII*, neomycin phosphotransferase gene; *NOS Ter*, terminator of neomycin phosphotransferase gene; MAR, the molecular structure of the matrix attachment region of tobacco; *CoYMV pro*, commelina yellow mottle virus promoter; *Cry3A*, bacillus thuringiensis insecticidal crystal protein gene; *CaMV35S pro*, cauliflower mosaic virus CaMV35S promoter; *AtADH* 5′-UTR, translation enhancer; *Cry1Ac*, bacillus thuringiensis insecticidal crystal protein gene; RB, right boundary of vector. **(B)** PCR confirmation of transgenic dual Bt toxin *Populus × euramericana* ‘Neva’ lines. M: DL2000 DNA Marker (From top to bottom, bands correspond to 2,000, 1,000, 750, 500, 250, and 100 bp); 1–16: sixteen rooting lines; CK−, non-transgenic control; CK+, positive control (plasmid N5).

Tissue culture seedlings of *Populus* × *euramericana* ‘Neva’ served as the transgenic receptor material. The plant expression vector N5 was constructed by the Forest Genetics and Breeding Laboratory of Hebei Agricultural University and maintained in *Agrobacterium tumefaciens* GV3101 (with kanamycin and rifampicin resistance). From the left border to the right border, the vector carried the selectable *nptII* marker gene and the dual Bt toxin genes *Cry3A* (GenBank accession number: M84650.1) and *Cry1Ac* (GenBank accession number: AF148644.1, with a small amount of base modification). A nuclear matrix attachment region (MAR, GenBank accession number: U67919.1) was present on the outside of the two Bt toxin genes. The T-DNA structure of the vector is shown in Figure 1A.

### Insects Used in Experiments

*Hyphantria cunea* belongs to Lepidoptera Arctiidae. Eggs were provided by Professor Min Chen of Beijing Forestry University. After hatching, the larvae were used in indoor feeding experiments.

*Micromelalopha troglodyta* belongs to Lepidoptera Notodontidae. Eggs were collected from Baoding, Hebei Province, China. After hatching, the larvae were used in indoor feeding experiments.

*Plagiodera versicolora* belongs to Coleoptera Chrysomelidae. Eggs and adults were collected from Baoding. After the eggs hatched, the first instar larvae were used in experiments. Non-transgenic poplar leaves were used to feed the larvae from the first to second instar, and the second instar larvae were used in experiments. Captured adults were tested directly.

*Anoplophora glabripennis* belongs to Coleoptera Cerambycidae. Adult insects were captured in Baoding. After mating, they were placed in insect rearing boxes (19 × 12.5 × 7.5 cm), and a small number of fresh leaves and one willow wood segment (15 cm in length and 5–8 cm in diameter) were placed in the boxes for adults to obtain nutrients and lay eggs. The wood segment was replaced every 3 days. After 1 week, the bark was removed and the eggs were placed in a culture dish (9 cm in diameter) with wet filter paper on the bottom. The eggs were reared in a dark environment at room temperature. After the eggs hatched, the larvae were used in indoor feeding experiments.

### Production of Transgenic Dual Bt Toxin *Populus* × *euramericana* ‘Neva’ Lines

#### *Agrobacterium*-Mediated Leaf Disk Genetic Transformation

The dual Bt toxin genes were integrated into the genome of *Populus* × *euramericana* ‘Neva’ using the *Agrobacterium-*mediated leaf disk method. Resistant lines were obtained after kanamycin screening. After propagation, the lines were domesticated and transplanted to the experimental field, watered regularly, and loosened the soil. Transformations were performed according to Liu et al. (2016) with the following modifications: 100 μM acetosyringone (AS) was added to the co-culture medium and the concentration of kanamycin in rooting medium was increased to 50 mg⋅L^–1^ to reduce the occurrence of false positives during screening.

#### Polymerase Chain Reaction (PCR) Detection of Exogenous Genes

In early June, the leaves of kanamycin-resistant lines and non-transgenic *Populus* × *euramericana* ‘Neva’ were collected from the experimental field and genomic DNA was extracted by an improved cetyltrimethylammonium bromide (CTAB) method (Wang and Fang, 2002). PCR detection was performed using specific primers for the *nptII*, *Cry1Ac*, and *Cry3A* genes (primers 103#, 105#, and 106#; Supplementary Table 1). The plasmid N5 was used as a positive control and non-transgenic *Populus* × *euramericana* ‘Neva’ plants served as a negative control. PCR reactions were performed according to Liu et al. (2016) with a slight modification: the annealing temperature for the *Cry3A* gene was 52°C. The theoretical sizes of the amplified fragments of the *nptII*, *Cry1Ac*, and *Cry3A* genes were 473, 546, and 667 bp, respectively. PCR product sizes were observed by 1% TAE agarose gel electrophoresis.

### Detection of Exogenous Gene Expression in Transgenic Lines

#### Fluorescence Quantitative PCR (FQ-PCR) Detection

In early July, three biological replicate samples of fully expanded young leaves from all transgenic lines and non-transgenic *Populus* × *euramericana* ‘Neva’ (CK) were collected from the field, frozen in liquid nitrogen, and stored at −80°C. Total RNA was extracted and reverse transcribed to produced first-strand cDNA using RNA extraction and reverse transcription kits (SENO, Zhangjiakou, Hebei, China) according to the manufacturer’s instructions. The FQ-PCR primers 176# and 1291# (amplified target fragments were 176 and 203 bp, respectively; Supplementary Table 1) were designed according to the full *Cry1Ac* and *Cry3A* nucleotide sequences using Primer Premier 6.0 (Premier Biosoft, Canada) software. Absolute FQ-PCR was performed using AceQ qPCR SYBR Green Master Mix (Vazyme Biotech Co., Ltd., Nanjing, China) and an Agilent Technologies Stratagene Mx3005P Real-Time PCR instrument (Agilent, United States) to determine the transcriptional expression levels of the exogenous genes. FQ-PCR was performed according to Liu et al. (2016).

#### Bt Toxin Detection in Transgenic Lines

The presence of Bt toxin proteins in the same samples described above in section of “Fluorescence quantitative PCR (FQ-PCR) detection” was detected using Bt-Cry1Ab/1Ac and Bt-Cry3A ELISA kits (Agdia, United States) according to the manufacturer’s instructions. The positive controls used were included in the kits, and the negative control was CK. The results were determined using a Bio-RAD Model 550 microplate reader (Bio-Rad). The toxin content was calculated as the amount of toxin in each gram fresh weight of leaves (ng⋅g^–1^ FW).

### Insect Resistance of Transgenic Lines

#### Lepidopteran Target Insect Feeding Experiment

From late July to early August, the top third or fourth fresh leaves of transgenic and CK lines were collected to conduct indoor feeding experiments on the first instar (L1) larvae of *H*. *cunea* and *M*. *troglodyta*. The petioles were inserted into humid flower mud (2 × 2 × 2 cm) to maintain freshness of the leaves. Thirty L1 larvae were placed on each leaf with a brush and placed in a breathable feeding bottle (6.5 cm in diameter at the bottom and 8 cm in height). The leaves and flower mud were replaced daily, and larval mortality was recorded. Each treatment was repeated three times. After the number of surviving larvae in the treatment and control groups stabilized (*H*. *cunea* were fed for 14 days and *M*. *troglodyta* were fed for 10 days), the experiment was terminated. The data were recorded and the relevant indexes were calculated according to the following formulas.

(1) $$Mortalityrate=\frac{numberofdeaths}{totalnumberoftestedinsects}\times 100\%$$

(2) $$\begin{array}{c} \mathrm{Corrected mortality rate}=\\ \;\frac{\left(\mathrm{mortality rate of transgenic lines}-\mathrm{mortality rate of control}\right)}{\left(1-\mathrm{mortality rate of control}\right)}\\ \;\times 100\% \end{array}$$

#### Coleopteran Target Insect Feeding Experiment

In mid-August, the top third or fourth fresh leaves of transgenic and CK lines were collected to conduct indoor feeding experiments on the first instar (L1) larvae, second instar (L2) larvae, and adults of *P*. *versicolora*, with 30, 20, and 10 insects, respectively, included in each replicate. Other feeding methods were the same as above. After feeding for 3, 20, and 22 d, respectively, the experiments were terminated. The data were recorded and the relevant indexes were calculated using the same methods as above.

In late August, six transgenic lines (1, 7, 9, 11, 14, and 15) and CK were selected to feed newly hatched *A*. *glabripennis* larvae. The weights of the newly hatched larvae were recorded using a milligram balance, and the larvae were transferred to a feeding box (25 mL) with soft tweezers. The xylem and leaves of tree segments were mixed and crushed to use as feed. The feeding boxes were numbered, each box contained one larva, and each treatment had ten replicates. All feeding boxes were placed in a dark environment at room temperature. The feed was replaced every other day, the mortality was recorded, and the surviving larvae were weighed. After feeding for 35 days, differences between treatments were significant and the experiment was terminated. Each insect was weighed and stored in a centrifuge tube at −80°C for use in subsequent enzyme activity determination and RNA-seq analyses. The mortality, weight increment, and rate of *A*. *glabripennis* larval growth inhibition were calculated according to formulas (1), (3), and (4).

(3) $$\begin{array}{c} Increment=measuredvalueattheendoftheexperiment\\ -measuredvalueatthebeginningoftheexperiment \end{array}$$

(4) $$\begin{array}{c} \mathrm{Growth inhibition rate}=\\ \;\frac{\left(\mathrm{average increment of control group}-\mathrm{average increment of treated group}\right)}{\mathrm{average increment of control group}}\\ \;\times 100\% \end{array}$$

#### Determination of Cellulase Activity in the Midgut of *A. glabripennis* Larvae

Cellulase is a complex enzyme, composed mainly of exo-1,4-β-D-glucanase (C1 enzyme), endo-1,4-β-D-glucanase (Cx enzyme), and β-1,4-glucosidase (β-glucosidase). In this study, the activities of Cx enzyme and β-glucosidase in the *A*. *glabripennis* midgut were determined. The *A*. *glabripennis* larvae stored at −80°C were thawed at 4°C for 4 h, and each treatment was repeated three times. The intestinal tract was dissected on ice, homogenized on ice in 2 mL cold acetic acid-sodium acetate buffer solution (pH 5.2, 0.1 mol⋅L^–1^), and centrifuged at 12,000 r⋅min^–1^ for 20 min at 4°C. The supernatant containing the isolated enzymes was stored at −30°C.

The cellulase components were determined by the 1, 3- dinitrosalicylic acid (DNS) method (Shi et al., 2011), and a glucose standard curve was drawn according to Sinegani and Emtiazi (2006). Cx enzyme activity was determined using 1% carboxymethyl cellulose (CMC) as the substrate, while β-glucosidase activity was determined with 1% salicylic acid as substrate according to the method of Li et al. (2011). Each treatment was repeated three times. Enzyme activity (μmol glucose⋅g^–1^ FW⋅h^–1^) was calculated according to the amount of reducing sugar (glucose) produced by enzymatic reaction per unit body weight (FW) per unit time (h).

### Transcriptome Sequencing

#### RNA Extraction, cDNA Library Preparation, and RNA-Seq Analyses

Based on the results of the feeding experiments and the cellulase activities of *A*. *glabripennis* larvae, the larvae that were fed with transgenic lines 7 and 15 and with CK were selected for RNA-seq analyses, and named treatment groups A, B, and CK, respectively. Larvae with the same growth vigor were selected for each treatment. Each treatment was repeated three times with one larva per replicate. Total RNA was extracted by the TRIzol method. The quality and concentration of RNA were determined using an Agilent 2100 Bioanalyzer (Agilent Technologies, Palo Alto, CA, United States) and a NanoDrop spectrophotometer (Thermo Fisher Scientific Inc.). Three cDNA libraries were constructed and after quality inspection, were sequenced using an Illumina HiSeq 2000 high-throughput sequencing platform.

#### Sequencing Quality Evaluation, Sequence Alignment, Screening, and Functional Annotation of Differentially Expressed Genes (DEGs)

Clean data were obtained after quality evaluation and preprocessing of sequencing data using FastQC (ver. 0.10.1) and Cutadapt (ver. 1.9.1) software, and compared against the *A*. *glabripennis* reference genome^1^ using Hisat2 (ver. 2.0.1) software. Gene annotation was performed according to the *A*. *glabripennis* reference genome annotation document^2^. Gene expression levels were calculated as fragments per kilobase of exon model per million reads mapped (FPKM) with Htseq (ver. 0.6.1) software, and the DEGs among samples were analyzed using DESeq2 (ver. 1.6.3) software. The formula used to calculate the fold-change (FC) between the two groups was log_2_FC = log_2_(treatment group/control group). A gene was designated as a DEG when | log_2_FC| > 1 and *P*-value < 0.05. A two-sample differential expression analysis method was applied to identify the number of DEGs, and the numbers of upregulated and downregulated genes were calculated according to the log_2_FC value. K-means hierarchical clustering software was used for cluster analyses of the expression patterns of all DEGs. Gene Ontology (GO) and Kyoto Encyclopedia of Genes and Genomes (KEGG) databases were used for functional annotation, classification of DEGs, and enrichment analyses of metabolic pathways. Heatmaps were generated using the among-samples normalized FPKM values as an indicator of gene expression values with the Pheatmap R package.

### Verification of DEGs With Real-Time Quantitative Reverse Transcription PCR (RT-qPCR)

Seven genes were randomly selected from the RNA-seq results. The glyceraldehyde-3-phosphate dehydrogenase (*GAPDH*, GenBank accession number: KU521365.1) gene served as the reference gene. The reliability of RNA-seq results was verified by RT-qPCR. Primers were designed using Primer Premier 6.0 software. The primer information is presented in Supplementary Table 1. The HiScript III RT SuperMix for qPCR (+gDNA wiper) reverse transcription kit (Vazyme Biotech Co., Ltd., Nanjing, China) was used for reverse transcription of the samples after sequencing. RT-qPCR analyses were conducted using AugeGreen qPCR Master Mix (US Everbright Inc., Suzhou, China) and the Agilent Technologies Stratagene Mx3005P Real-Time PCR instrument (Palo Alto, CA, United States) according to Ren et al. (2020). Each sample was repeated three times. Relative expression was calculated by the 2^–ΔΔCT^ method.

### Statistical Analyses

Excel 2016 (Microsoft, Inc., Redmond, WA, United States) and Data Processing System (DPS) 7.05 (China) software were used to consolidate and analyze the data. The data were analyzed for each treatment using one-way analysis of variance (ANOVA), and significance was tested using Duncan’s multiple range test (*P* < 0.05). Excel 2016 software was used to create tables and OriginPro 2018 (OriginLab Corporation, Northampton, MA, United States) software was used to create drawings.

## Results

### Production of Dual Bt Toxin *Populus* × *euramericana* ‘Neva’ Transgenic Lines and Confirmation of Exogenous Genes by PCR

*Agrobacterium tumefaciens* harboring the transformation construct containing the *Cry1Ac* and *Cry3A* genes was inoculated into incisions in leaf disks resulting in production of green buds in some of the incisions. Kanamycin-resistant buds were transferred to rooting medium containing kanamycin. After 5–7 days, most of the resistant buds grew roots. The shoots of the rooting plants were transferred to differentiation selection medium to propagate. Then kanamycin-resistant shoots were induced to root, the rooted plantlets were transplanted into small flowerpots, and the plants were subsequently transplanted to the experimental field. In total, 16 transgenic dual Bt toxin *Populus* × *euramericana* ‘Neva’ kanamycin-resistant rooted lines with were obtained. Presence of the *Cry1Ac*, *Cry3A*, and *nptII*, transgenes in the lines was confirmed by PCR followed by gel electrophoresis (Figure 1B). Bands corresponding to the three exogenous genes were amplified in all transgenic lines and the positive control, but not detected in the negative control, indicating that the exogenous genes had integrated into the *Populus* × *euramericana* ‘Neva’ genome.

### Transgene Expression Levels in Transgenic Lines

**FIGURE 2:**
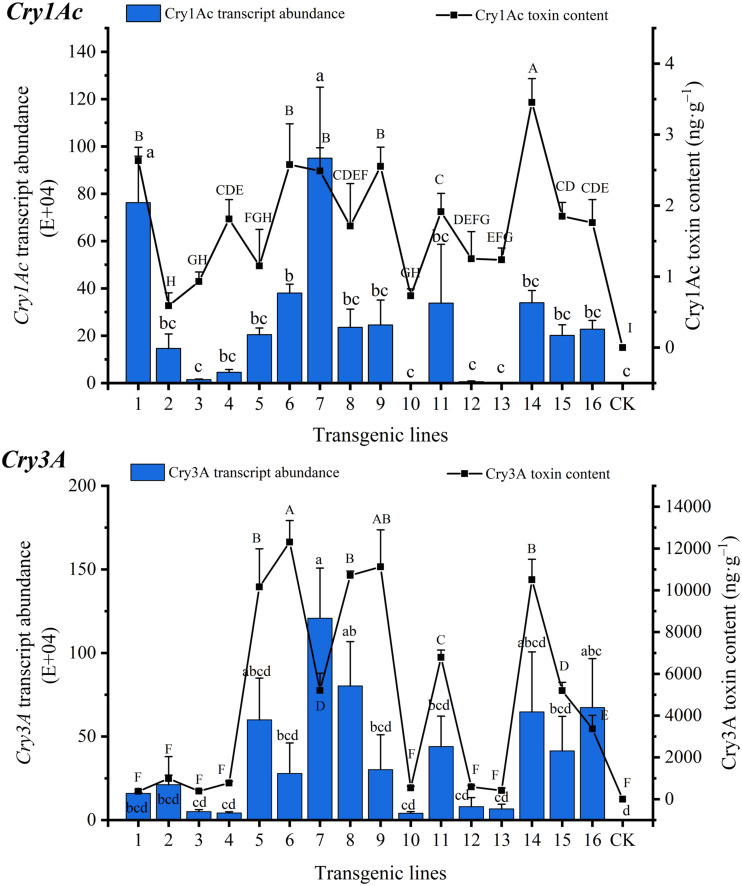
Transgene transcript abundance and Bt toxin protein content in transgenic lines. Lowercase letters indicate multiple comparisons of the transcript abundance of the transgenic lines (*P* < 0.05), and capital letters indicate multiple comparisons of the Bt toxin protein contents of the transgenic lines (*P* < 0.05).

The Bt toxin transgene transcript abundances and protein contents of transgenic lines are shown in Figure 2. The *Cry1Ac* transcript abundances ranged from 1.15E+03 to 9.50E+05, with the highest expression in line 7 and the lowest expression in line 13. The *Cry3A* transcript abundances ranged from 4.06E+04 to 1.21E+06, with the highest expression in line 7 and the lowest expression in line 10. The fluorescence signals corresponding to the two genes were not detected in CK. The *Cry1Ac* and *Cry3A* transcript abundances differed among the lines, with significant differences found between some lines. The coefficients of variation of *Cry1Ac* and *Cry3A* transcript abundance were 1.014 and 0.865, respectively. The correlation coefficient of the two genes was 0.634 (*P* < 0.01) (Supplementary Table 2).

Bt toxin protein expression was detected in all transgenic lines, but there were differences among the lines, some of which were significant. Cry1Ac toxin content ranged from 0.591 ± 0.182 to 3.453 ± 0.336 ng⋅g^–1^ FW, with the highest content in line 14 and the lowest content in line 2, while Cry3A toxin content ranged from 373.485 ± 77.232 to 12,308.543 ± 1,030.893 ng⋅g^–1^ FW, with the highest content in line 6 and the lowest content in line 1. The Cry3A toxin content was much higher than that of Cry1Ac. The coefficients of variation of Cry1Ac and Cry3A toxin content were 0.430 and 0.906, respectively, and the correlation coefficient was 0.554 (*P* < 0.05) (Supplementary Table 2) indicating a significant correlation. The correlation coefficients between the transcript abundances of *Cry1Ac* and *Cry3A* and the corresponding toxin proteins were 0.644 (*P* < 0.05) and 0.535 (*P* < 0.05), respectively (Supplementary Table 2), indicating a significant correlation between the transcript abundance and the toxin protein content.

### Resistance of Transgenic Lines to Target Insects

#### Resistance of Transgenic Lines to Lepidopteran Pests

**FIGURE 3:**
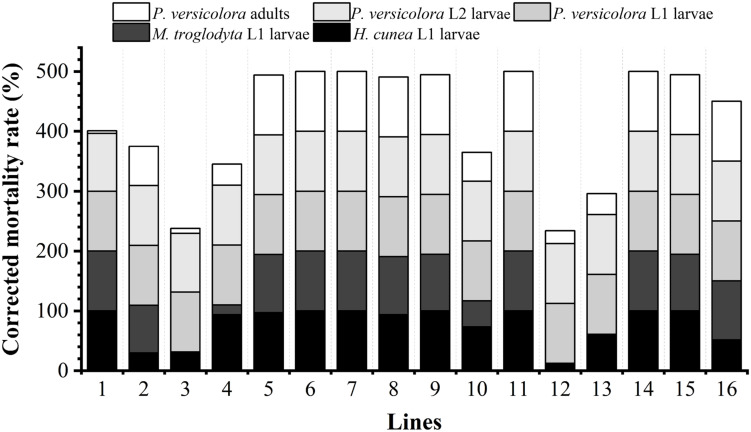
Resistance of transgenic lines to target insects. *M*. *troglodyta* L1 larvae were not fed lines 3, 12, or 13.

The lethal effects of the transgenic lines on the L1 larvae of *H*. *cunea* and *M*. *troglodyta* are shown in Figure 3 and photographs of feeding experiments are presented in Supplementary Figure 1. Insect resistance levels differed among the transgenic lines. The corrected mortality rates of *H*. *cunea* L1 larvae fed transgenic lines 1, 6, 7, 9, 11, 14, and 15 reached 100%, while those of larvae fed lines 4, 5, and 8 were greater than 93%. The other lines caused lower mortality rates, with line 12 causing the lowest mortality (12.50%). The corrected mortality rates of *M*. *troglodyta* L1 larvae fed transgenic lines 1, 6, 7, 11, and 14 reached 100%, and those of larvae fed lines 5, 8, 9, 15, and 16 were greater than 94%. The mortality rates caused by other lines were lower, with line 4 causing the lowest mortality (16.21%). The transgenic lines exhibited resistance to the L1 larvae of *H*. *cunea* and *M*. *troglodyta*, with lines 1, 6, 7, 11, and 14 exhibiting the highest levels of resistance. The correlation coefficients between Cry1Ac toxin content and the corrected mortality rates of *H*. *cunea* and *M*. *troglodyta* L1 larvae were 0.668 (*P* < 0.01) and 0.419 (*P* < 0.05), respectively (Supplementary Table 2), indicating extremely significant and significant correlations, respectively.

#### Resistance of Transgenic Lines to *P. versicolora* (Coleoptera)

The lethal effects of the transgenic lines on the L1 and L2 larvae and adults of *P*. *versicolora* are shown in Figure 3, and photographs of feeding experiments are shown in Supplementary Figure 1. The levels of insect resistance differed among the transgenic lines, with some differences being significant. The corrected mortality rates of *P*. *versicolora* L1 larvae after feeding reached 100% for all of the transgenic lines. The corrected mortality rates of *P*. *versicolora* L2 larvae reached 100% for most of the transgenic lines with only line 2 causing a slightly lower mortality rate of 96.21%. The corrected mortality rates of adults reached 100% for most lines, and the corrected mortality rates of lines 1, 2, 3, 4, 10, 12, and 13 ranged from 4.17% to 66.0%, with lines 1 and 3 exhibiting the least resistance. On the whole, lines 5, 6, 7, 8, 9, 11, 14, 15, and 16 exhibited high resistance to L1 and L2 larvae and adults of *P*. *versicolora*. The correlation coefficients between Cry3A toxin content and corrected mortality rates of L1 and L2 larvae and adults were 0 and 0.363 (*P* < 0.05), and 0.810 (*P* < 0.01), respectively (Supplementary Table 2). The latter two correlation coefficients were significant and extremely significant, respectively.

Based on the observed lethal effects of the transgenic lines on *H*. *cunea*, *M*. *troglodyta*, and *P*. *versicolora*, lines 5, 6, 7, 8, 9, 11, 14, and 15 exhibited strong insect resistance, while line 1 showed poor resistance against *P*. *versicolora* adults.

#### Inhibitory Effects of Transgenic Lines on *A. glabripennis* Larval Growth and Development

**FIGURE 4:**
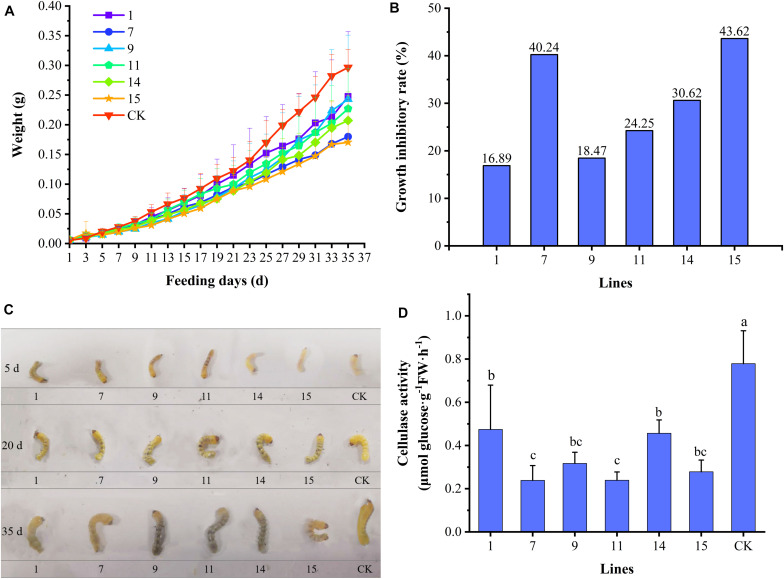
Growth indexes of *A*. *glabripennis* larvae. **(A)** Variation in average body weights of *A*. *glabripennis* larvae fed different lines. **(B)** Rates of growth inhibition in *A*. *glabripennis* larvae fed different transgenic lines. **(C)** Comparison of the larvae fed the different lines. **(D)** Cellulase activities in the midguts of larvae fed different transgenic lines.

During feeding on the transgenic lines by *A*. *glabripennis* larvae, only one larva died on lines 7, 9, 11, and 15 (10% mortality). The variation in the average weights of *A*. *glabripennis* larvae is shown in Figure 4A. At the beginning of feeding, changes in larval weight were essentially the same among the larvae. After 9 days of feeding, differences began to appear and gradually became obvious. After 23 days, the weights of the larvae fed the control increased rapidly, but those of the larvae fed the transgenic lines increased relatively slowly. On day 35, the weights of the larvae showed hierarchical differentiation. Larvae fed CK had the highest weights, followed by those fed lines 1, 9, 11, and 14, and the larvae with the lowest weights were those fed lines 7 and 15. There were differences in the weight increment of *A*. *glabripennis* larvae fed the various transgenic lines. The growth inhibition rates of *A*. *glabripennis* larvae fed transgenic lines are shown in Figure 4B. The growth inhibition rate reached more than 40% for the larvae fed lines 7 and 15, indicating strong inhibition, while the growth inhibition rates of the other four lines ranged from 16.89% to 30.62%. A comparison of larval growth and development is shown in Figure 4C. The larvae fed transgenic lines were slightly blackened, while those fed CK were white. On day 35, the larvae fed lines 7 and 15 were significantly smaller than those fed CK, and were curled and exhibited lower activity, indicating that these two transgenic lines had strong inhibitory effects on the growth and development of *A*. *glabripennis* larvae, while there was little difference between the other lines and CK. Cellulase activity levels in the larval midgut are shown in Figure 4D. The cellulase activities were significantly lower in the midguts of larvae fed the transgenic lines than in those fed CK, and the cellulase activities in the larvae fed lines 7, 11, and 15 were less than 36% of those fed CK. There was a significant correlation coefficient of 0.789 (*P* < 0.05) between cellulase activity and body weight (Supplementary Table 2). These results suggest that Bt toxin can indirectly affect the growth and development of *A*. *glabripennis* larvae by inhibiting cellulase activity in the midgut.

### Transcriptional Response of *A. glabripennis* Larvae to Bt Toxin

After quality control to remove sequencing junctions, contamination, and low-quality data, 59.5 Gb of clean data were obtained from RNA-seq analyses (Supplementary Table 3). The mapped rate of each sample was > 81%, the GC content ranged from 44.40% to 47.17%, and the Q30 was > 93% (Supplementary Table 3), indicating that the sequencing data were suitable for further analyses.

#### Identification of DEGs and Hierarchical Cluster Analyses

**FIGURE 5:**
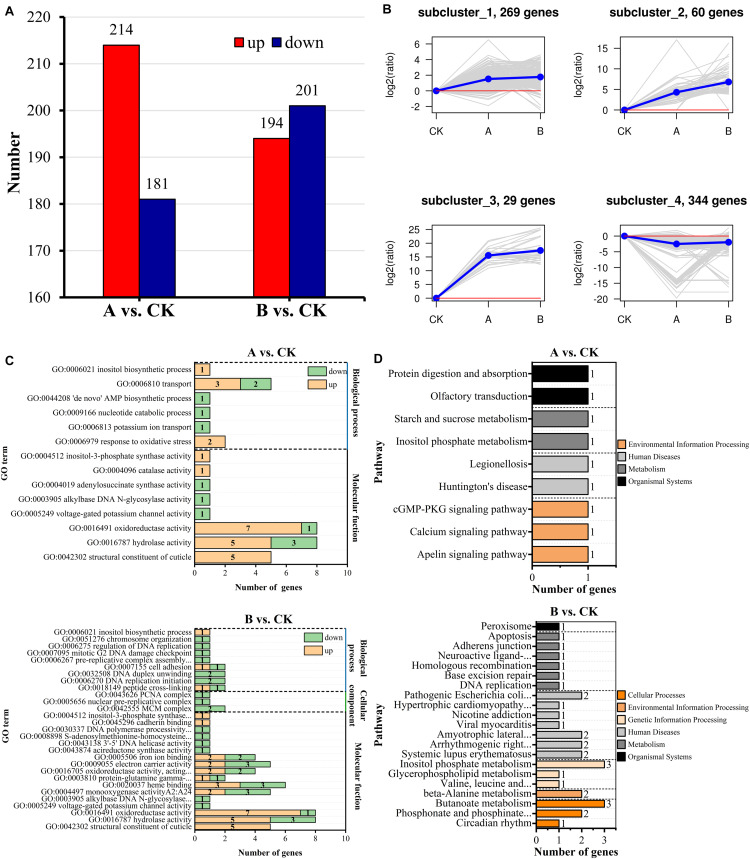
Identification, clustering, and functional enrichment analyses of DEGs. **(A)** Statistics of the number of DEGs. **(B)** Analyses of expression patterns. **(C)** Significant enrichment map of GO terms. **(D)** Significant enrichment map of KEGG pathways.

To explore the mechanism of Bt toxin inhibition of *A*. *glabripennis* larval growth and development, DEGs were identified meeting criteria of *P* < 0.05 and | log_2_FC| > 1. The statistical results are shown in Figure 5A. Comparison of treatment group A with control group CK (A vs. CK) identified 395 DEGs of which 181 genes were downregulated and 214 genes were upregulated. There were also 395 DEGs identified in the comparison of treatment group B compared with CK (B vs. CK), including 201 downregulated genes and 194 upregulated genes. There were 88 DEGs shared between the two comparisons, of which 86 DEGs had consistent expression patterns, while those of 2 DEGs differed between the comparisons (Supplementary Table 4).

K-means hierarchical clustering analyses were performed on all of the DEGs in the two comparative analysis groups. The results clustered into four expression patterns (Figure 5B). Subcluster_1, subcluster_2, subcluster_3, and subcluster_4 contained 269, 60, 29, and 344 DEGs, respectively. Among them, subcluster_1, subcluster_2, and subcluster_3 showed gene expression levels that were higher in treatment groups A and B than in CK, and the changes in gene expression were more significant in treatment group B. By contrast, subcluster_4 showed gene expression levels that were lower in treatment groups A and B than in CK, and the gene expression changes were more significant in treatment group A.

#### GO Functional Enrichment and KEGG Annotation of Metabolic Pathways

Functional annotation of DEGs was performed according to the GO database (Figure 5C). DEGs of A vs. CK belonged to 14 subclasses in molecular functions and biological processes (*Q*-value < 0.05) and were mainly enriched in structural constituent of cuticle, hydrolase activity, response to oxidative stress, oxidoreductase activity, catalase activity, and inositol biosynthesis process. DEGs of B vs. CK belonged to 24 subclasses in biological processes, cellular components, and molecular functions, which were mainly enriched in monooxygenase activity, heme binding, oxidoreductase activity, acireductone synthase activity, cadherin binding, and inositol biosynthetic process. The types of GO terms enriched for among the DEGs differed between treatment groups A and B.

Functional annotation and pathway analyses were performed according to the KEGG database. Five DEGs of A vs. CK were enriched and annotated to 10 metabolic pathways, and 21 DEGs of B vs. CK were enriched and annotated to 83 metabolic pathways. Based on the enrichment results (*Q*-value < 0.05) (Figure 5D), there were nine significantly enriched pathways in A vs. CK, mainly including protein digestion and absorption, olfactory transduction, inositol phosphate metabolism, starch and sucrose metabolism, and calcium signaling pathway. There were 21 significantly enriched pathways in B vs. CK, mainly including phosphonate and phosphinate metabolism, DNA replication, adherens junction, amyotrophic lateral sclerosis, peroxisome, and inositol phosphate metabolism. The inositol phosphate metabolism pathway was shared by the two comparative analysis groups.

#### Transcription Factor (TF) Analyses

**FIGURE 6:**
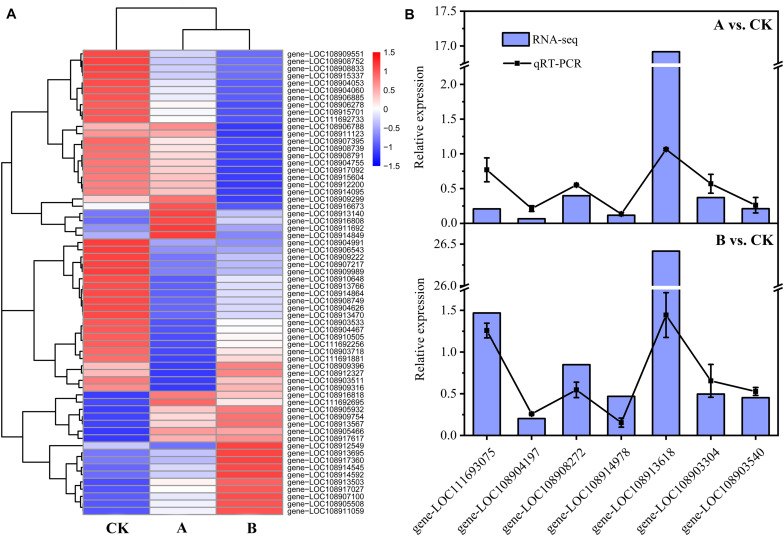
Heat map of TF expression and RT-qPCR validation. **(A)** Heat map of TF expression. **(B)** RT-qPCR analyses to confirm expression patterns indicated by RNA-seq analyses. Genes were randomly selected for RT-qPCR. The RT-qPCR results are presented as the mean average of 2^–ΔΔCT^ values. The RNA-seq results are presented as the Fold Change. gene-LOC111693075, sugar transporter ERD6-like 4; gene-LOC108904197, probable transaldolase; gene-LOC108908272, cathepsin B-like; gene-LOC108914978, uncharacterized LOC108914978; gene-LOC108913618, uncharacterized LOC108913618; gene-LOC108903304, glutathione S-transferase-like; gene-LOC108903540, endoglucanase.

Changes in the expression patterns of TFs can affect the expression levels of regulatory genes. A total of 30 TFs (20 downregulated, 10 upregulated) were identified among the DEGs of A vs. CK. They belonged to 11 TF families, mainly including zf-C2H2 (10), TF_others (6), ZBTB (3), bHLH (2), Homeobox (2), and MBD (2). A total of 39 TFs (24 downregulated, 15 upregulated) were identified among the DEGs of B vs. CK. They belonged to 12 TF families, mainly including zf-C2H2 (10), Homeobox (5), bHLH (4), TF_others (4), MYB (3), TF_bZIP (3), ZBTB (3), and zf-LITAF-like (3). In the two comparative analysis groups, the number of downregulated TFs was greater than the number of upregulated TFs. The expression pattern of each TF is shown in Figure 6A and Supplementary Table 5. It is speculated that the expression of regulatory genes in *A*. *glabripennis* larvae is affected by the differential expression of TFs, and plays a role in the insect immune response to Bt toxin.

#### RT-qPCR Verification

Seven genes were randomly selected for RT-qPCR analyses to verify the accuracy of the RNA-seq results. The results of comparison of the RT-qPCR results with the RNA-seq data are shown in Figure 6B. In the two comparative analysis groups, the RT-qPCR and RNA-seq results for six genes showed little difference, while the results for one gene (gene-LOC108913618) differed greatly. However, the expression patterns of the genes as determined by RT-qPCR and RNA-seq were the same, which demonstrated the reliability of the transcriptome data obtained in this study.

### Identification of Genes in *A. glabripennis* Larvae That Are Differentially Expressed in Response to Bt Toxin

**FIGURE 7:**
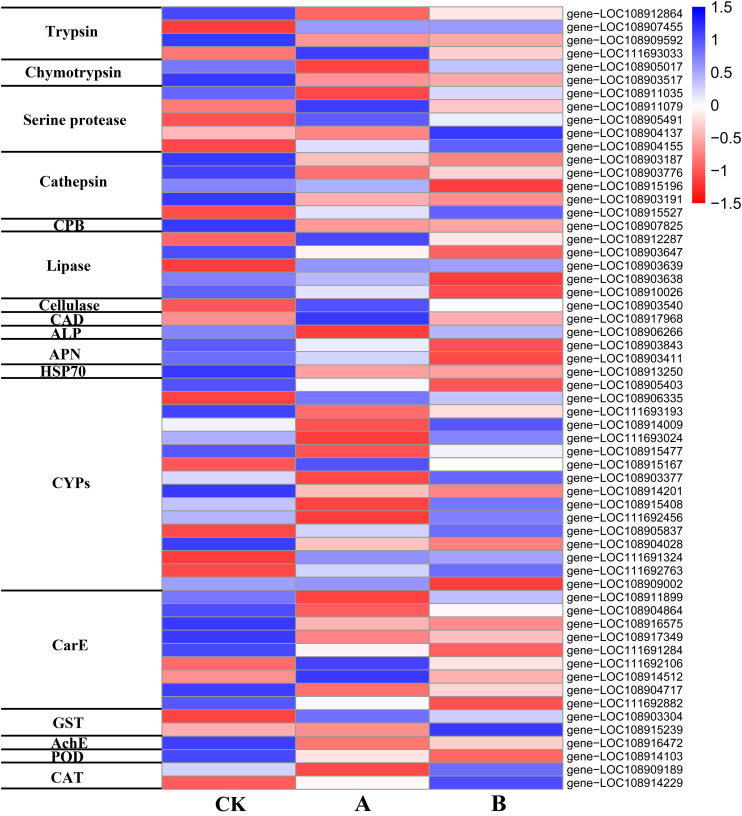
Heat map of DEG expression.

Based on the gene IDs of the DEGs, annotation information was retrieved from NCBI, and the DEGs related to Bt toxin mechanism, digestive enzymes, detoxification enzymes, and protective enzymes of *A*. *glabripennis* larvae were identified (Supplementary Table 6). Based on the expression level (FPKM) data, a heat map of the 59 identified DEGs was drawn (Figure 7), and the expression differences in genes related to the Bt toxin response between the two transgenic lines and CK were analyzed.

#### Bt Prototoxin Activation- and Digestion-Related Proteases

Trypsin and chymotrypsin are important digestive enzymes and play a critical role in the activation of Bt prototoxin. In this study, the expression of genes encoding prototoxin activation-related enzymes changed in *A*. *glabripennis* larvae fed poplar feed containing Cry3A toxin. Four trypsin-related genes (two upregulated, two downregulated) and two chymotrypsin-related genes (both upregulated) were identified among the DEGs of A vs. CK (Figure 7 and Supplementary Table 6), but were not found in the DEGs of B vs. CK. If the conditions of DEG identification were set as *P* < 0.1 and | log_2_FC| > 1, two trypsin-related genes (one upregulated, log_2_FC = 1.237 and one downregulated, log_2_FC = −1.128) and two chymotrypsin-related genes (upregulated, log_2_FC = 1.236 and 1.056) were identified.

After food reaches the midgut, a variety of digestive enzymes participate in digestion and absorption. In addition to the two serine proteases, trypsin and chymotrypsin, mentioned above, other serine proteases, cathepsin, and carboxypeptidase, as well as lipase, and cellulase are also involved. Three serine protease-related genes (one upregulated, two downregulated), three cathepsin-related genes (two cathepsin L and one cathepsin B, upregulated), three lipase-related genes (one upregulated and two downregulated), and one cellulase-related gene (endoglucanase, downregulated) (Figure 7 and Supplementary Table 6) were identified among DEGs of A vs. CK. Two serine protease-related genes (downregulated), four cathepsin-related genes (two cathepsin L and one cathepsin B, upregulated; one cathepsin L, downregulated), one carboxypeptidase B gene (upregulated), and three lipase-related genes (two upregulated, one downregulated) were identified among the DEGs of B vs. CK (Figure 7 and Supplementary Table 6). After *A*. *glabripennis* larvae were fed poplar feed containing Bt toxin, the expression of various digestive enzymes changed, which may play a role in the insect immune response to Bt toxin.

#### Potential Bt Toxin-Binding Receptor Protein-Related DEGs

After Bt toxin is hydrolyzed and toxic peptide molecules are released, they must bind with specific receptors to perform their function. After the *A*. *glabripennis* larvae were fed poplar feed containing Cry3A toxin, the expression of genes encoding potential Bt toxin-binding receptors changed significantly (Figure 7 and Supplementary Table 6). Three Bt toxin-binding receptor-related genes were identified among the DEGs of A vs. CK, including one cadherin (CAD) gene (downregulated), one alkaline phosphatase (ALP) 4-like gene (upregulated), and one HSP70 gene (upregulated). Three toxin-binding receptor-related genes were identified among the DEGs of B vs. CK, including two aminopeptidase N (APN)-related genes (upregulated) and one HSP70 gene (upregulated).

#### Detoxification Enzyme-Related DEGs

Cytochrome P450 monooxygenase (CYP), carboxylesterase (CarE), and glutathione S-transferase (GST) are three detoxification enzymes that play key roles in resistance metabolism in insects. In addition, acetyl cholinesterase (AchE) is a detoxification enzyme that plays an important role in decomposing exogenous toxins and maintaining normal physiological metabolism in insects. In this study, the expression levels of detoxification enzyme-related genes changed significantly in *A*. *glabripennis* larvae fed poplar feed containing Cry3A toxin (Figure 7 and Supplementary Table 6). Eleven CYP-related genes (nine upregulated, two downregulated), seven CarE-related genes (five upregulated, two downregulated), one GST gene (downregulated), and one AchE gene (upregulated) were identified among the DEGs of A vs. CK. Seven CYP-related genes (three upregulated, four downregulated), five CarE-related genes (upregulated), one GST gene (downregulated), and one AchE gene (upregulated) were identified among the DEGs of B vs. CK. Most of the detoxification enzyme-related genes identified in this study were upregulated, indicating that the detoxification metabolic mechanism was activated after *A*. *glabripennis* larvae fed on plant materials containing Bt toxin.

#### Protective Enzyme-Related DEGs

The expression of protective enzymes in *A*. *glabripennis* larvae fed poplar feed containing Cry3A toxin also changed (Figure 7 and Supplementary Table 6). One peroxidase (POD)-related gene (upregulated) and one catalase (CAT)-related gene (upregulated) were identified among the DEGs of A vs. CK. One POD-related gene (upregulated) and one CAT-related gene (downregulated) were identified among the DEGs of B vs. CK.

## Discussion

### Exogenous Gene Expression and Effect on Insect Resistance in Dual Bt Toxin *Populus* × *euramericana* ‘Neva’ Transgenic Plants

The use of combinations of multiple transgenes to improve plant traits is a new trend in transgenic plant research. Dong et al. (2014) and Zhou et al. (2020) introduced several exogenous genes in the same expression vector into poplar and obtained transgenic plants with multiple resistance traits. In this study, *Cry1Ac* and *Cry3A* genes were simultaneously transformed into *Populus* × *euramericana* ‘Neva’ and 16 transgenic lines were obtained through kanamycin screening and confirmed by PCR. The dual Bt toxin genes were expressed at both the transcriptional and translational levels. The Cry3A toxin content was much higher than that of Cry1Ac toxin, similar to previous research results (Dong et al., 2014; Zhou et al., 2020). This difference may be related to gene structure and promoter type used. In this study, most of the transgenic lines had strong insecticidal effects against *H*. *cunea* and *M*. *troglodyta* L1 larvae, and *P*. *versicolora* L1 and L2 larvae and adults, but poor toxicity against *A*. *glabripennis* larvae, although their growth and development were significantly inhibited. The levels of resistance of different transgenic lines to the same target insect also differed, which may be related to transgene insertion position and copy number.

Our laboratory previously performed studies on transgenic *Populus* × *euramericana* ‘Neva’ expressing dual *Bt* and salt-tolerance multi-gene. The transgenic lines obtained exhibited high resistance to *P*. *versicolora* larvae, but poor resistance to *H*. *cunea* larvae (Yang et al., 2016; Liu et al., 2016). Compared with previous studies, the insecticidal activity of the transgenic lines against *H*. *cunea* L1 larvae was improved, and the corrected mortality rates of some lines reached 100%, which may be related to adding an *AtADH* 5′-UTR enhancer upstream of the *Cry1Ac* gene. The *AtADH* 5′-UTR enhanced the expression of GUS by 150- and 87-fold in tobacco (*Nicotiana tabacum*) and *Arabidopsis thaliana*, respectively (Sugio et al., 2008). However, there were no significant differences in the insecticidal effects on *P*. *versicolora* L1 or L2 larvae. In this study, by contrast, the corrected mortality rates of *P*. *versicolora* adults fed transgenic lines reached as high as 100%, while the corrected mortality rates of *P*. *versicolora* third instar larvae were only 81.71% of that observed by Liu et al. (2016). The improved insecticidal effects of *Cry3A* may be related to the type of promoter used or the order of genes in the T-DNA. CoYMV has shown even greater activity than CaMV35S in tobacco and maize suspension cells (Medberry et al., 1992). Qiu et al. (2017) found that in tobacco transformed by dual Bt genes, the sequence and orientation of Bt gene expression cassette in the T-DNA could affect the expression content of Bt gene.

In this study, the insecticidal effects of individual transgenic lines against Lepidopteran *H*. *cunea* and *M*. *troglodyta* larvae differed, which may be related to the sensitivity of the insects to Bt toxin. The sensitivity to Bt toxin differs among insect species, possibly due to their different enzyme systems (Wang et al., 2018). In this study, the corrected mortality rates of *P*. *versicolora* larvae and adults fed transgenic lines 7, 9, 11, and 15 reached 100%, while those of newly hatched *A*. *glabripennis* larvae were only 10%, indicating that the leaf beetles were more sensitive to Bt toxin than longicorn beetles. Compared with CK, the transgenic lines significantly inhibited the growth and development of *A*. *glabripennis* larvae. The midgut cellulase activities of the *A*. *glabripennis* larvae fed transgenic lines were lower than those fed CK, and there was a significant correlation between the cellulase activity and the body weight of the larvae, indicating that Bt toxin affected the ability of the larvae to digest cellulose by inhibiting cellulase activity, thus inhibiting larval growth and development.

In this study, transgenic lines with high resistance to Lepidopteran and Coleopteran pests were produced. These lines provide new materials for developing transgenic *Populus* × *euramericana* ‘Neva’ plants expressing insect-resistance genes. Whether exogenous genes can be stably expressed in transgenic plants is an important factor affecting their application. The insect resistance of some transgenic poplar lines with high Bt toxin content has been observed to decrease with increasing tree age (Ren et al., 2018). The reason for this phenomenon may be that external environmental conditions and plant growth and development affect the expression and stability of exogenous genes through inactivation and silencing (Xia et al., 2000). At the same time, high Bt toxin gene expression may affect the growth and development of the transgenic plants (Ren et al., 2017). In future studies, the growth and development of dual Bt toxin *Populus* × *euramericana* ‘Neva’ transgenic plants and the stability of exogenous gene expression and insect resistance will be examined.

### Transcriptional Response of *A. glabripennis* Larvae to Bt Toxin

The expression of various genes in *A*. *glabripennis* larvae changed significantly after feeding on poplar feed containing Bt toxin. These genes may be involved in the immune response of *A*. *glabripennis* larvae to Bt toxin.

The activation of Bt prototoxin is a complex process in which trypsin and chymotrypsin play an important role. The activities of trypsin and chymotrypsin were increased by feeding the fifth instar larvae of *Helicoverpa armigera* (Lepidoptera) with Cry1Ac prototoxin for 12 h (Wei et al., 2016). However, compared with the control, the expression of trypsin-like gene was downregulated in *Tenebrio molitor* (Coleoptera) fed with Cry3Aa for 24 h (Oppert et al., 2012). This may be related to species and Cry toxin types. In this study, two trypsin and two chymotrypsin-related genes were upregulated and two trypsin genes were downregulated in A vs. CK, similar to the results of Yang et al. (2018) who examined the transcriptional response of *Cnaphalocrocis medinalis* (Lepidoptera) to Cry1C.

Serine protease is an important protease in the digestive and innate immune systems of insects, and changes in its expression may be related to insect resistance to Bt toxin (Yang et al., 2018). In this study, the differential expression of serine protease may be involved in the activation or degradation of Bt toxin. Studies have shown that cysteine and aspartic proteases are the main digestive enzymes of Coleoptera (Wu et al., 2016). After the third instar larvae of *Monochamus alternatus* (Coleoptera) were treated orally with Bt toxin insecticides, expression of the cathepsin L (cysteine protease) gene in the midgut was downregulated. It is speculated that Bt toxin may be toxic to *M*. *alternatus* by disrupting its development (Lin et al., 2016). After ingestion of eCry3.1Ab, the expression of a cathepsin B-like (cysteine protease) gene in susceptible strains of western corn rootworm was significantly increased (Zhao et al., 2019). In this study, cathepsin L and cathepsin B were differentially expressed in *A*. *glabripennis* larvae that fed on dual Bt toxin *Populus* × *euramericana* ‘Neva’ transgenic plants and they were mainly upregulated. Differences between this study and others may be related to the different species examined or the complex regulatory mechanism of Bt toxin. Cellulase is an important digestive enzyme that allows *A*. *glabripennis* to digest cellulose and obtain nutrients, and plays an important role in *A*. *glabripennis* growth and development. gene-LOC108903540, annotated as endoglucanase gene, performs the functions of carbohydrate metabolism process (go:0005975) and cellulase activity (go:0008810). In this study, the endoglucanase gene was differentially expressed in A vs CK, but not in B vs CK due to *P* > 0.05. However, the expression of endoglucanase gene decreased in both of them, and the cellulase activity in the midgut of longicorn larvae was significantly lower than that in the control. The growth and development of larvae in A and B were also significantly inhibited, indicating that Bt toxin could affect the growth and development of *A. glabripennis* larvae by inhibiting the cellulase activity.

At present, there are no reports of CAD, ALP, or APN acting as Bt toxin-binding receptors in *A*. *glabripennis* larvae. This study provides valuable information for further studies to determine whether these proteins are Bt toxin receptors in *A*. *glabripennis* larvae. Contreras et al. (2013) found a type of cadherin-like Cry3Aa receptor in *Tribolium Castanea* (Coleoptera: Tenebrionidae) and confirmed through gene silencing that the cadherin-like protein was a representative Cry receptor. In this study, one *CAD* gene was downregulated in A vs. CK, which was similar to the response of western corn root worm to Cry3Bb1 (Rault et al., 2018), which may be related to the pore formation model. Both APN and ALP are glycosylphosphatidylinositol (GPI)-anchored proteins, which play a role in the process of membrane insertion and pore formation. Many studies have shown that mutations of *APN1* or downregulated expression of *APN1* and *ALP2* are associated with Bt toxin resistance (Zhang et al., 2009; Tiewsiri and Wang, 2011), but no studies have reported that the upregulated expression of *APN1* or *ALP2* is associated with Bt toxin resistance. In this study, APN- and ALP-related genes were upregulated, which may show their own enzyme activities and play a role in their respective metabolism.

CYPs, CarE, and GST are important enzymes that play a central role in detoxification and metabolism of exogenous substances in insect systems. Several genes of the CYP6 family are related to resistance in insects, and CYP6 gene expression is upregulated as the main mechanism of resistance (Kim et al., 2017; Lee et al., 2018). In this study, CYP6, CYP4, CYP 12, and other CYP family genes were identified, and most of them were upregulated. They perform multiple functions such as oxidoreductase activity, metal ion binding, and binding (Supplementary Table 6), similar to the results of Behrens et al. (2014) in *Tribolium castaneum*. The CarE detoxification enzyme is involved in various functions including digestion and detoxification in *A*. *longicornis* (Coleoptera) (Han et al., 2019). In this study, most CarE-related genes were upregulated, and may play a certain role in detoxification during the immune response to Bt toxin. Glutathione S-transferases (GSTs) are a class of multifunctional detoxification enzymes, which are involved in the metabolism of many endogenous and exogenous toxic substances and the formation of drug resistance (Li et al., 2007). Studies have shown that a high level of GST expression is related to the insecticide resistance mechanism of insects (Bass and Field, 2011). In addition, GSTs can also protect cells from oxidative damage and intercellular transport of hormones, endogenous metabolites and exogenous compounds (Feng et al., 1999). In this study, gene-LOC108903304, annotated as glutathione S-transferase-like gene, was downregulated in A vs CK and B vs CK, indicating that Bt toxin may affect the detoxification and intercellular transport of toxin and larval resistance by affecting the expression of GST. The upregulated expression of the most detoxification enzymes indicates the induction of a metabolic detoxification mechanism in *A*. *glabripennis* larvae after feeding on poplar feed containing Bt toxin to reduce its toxicity.

When insects feed on Bt toxin, the balance of protective enzymes may be disrupted, potentially leading to excessive and damaging levels of O^2–^. Insects can protect and maintain normal functions by increasing the activities of protective enzymes, thus producing resistance to Bt toxin (Zhang Z. et al., 2011). Yang et al. (2018) found that POD gene expression patterns were both upregulated and downregulated, while CAT gene expression was downregulated in the transcriptional response of *C. medinalis* to Cry1C. In this study, POD gene expression was upregulated, while changes in CAT gene expression were not consistent between the two groups. Many studies have shown that increased POD expression is an emergency response to Bt toxin (Guo et al., 2011; Zhang Z. et al., 2011).

In conclusion, this study obtained dual Bt toxin *Populus* × *euramericana* ‘Neva’ transgenic lines with high insect resistance, which provides new materials for developing insect-resistant poplars. *A*. *glabripennis* larvae responded to Bt toxin by regulating the expression of genes related to Bt prototoxin activation, digestive enzymes, binding receptors, detoxification enzymes, and protective enzymes, which lays a theoretical foundation for establishing resistance to *A*. *glabripennis* in poplar, and provides a basis for exploring the mechanism of Bt toxin against Cerambycidae insects.

## Data Availability Statement

The original contributions presented in the study are publicly available. This data can be found here: The *A. glabripennis* larvae transcriptome data have been uploaded to Sequence Read Archive (https://www.ncbi.nlm.nih.gov/sra) under Bioproject PRJNA691113.

## Author Contributions

YR conducted the experiments, analyzed the data, and wrote the manuscript. XZ conducted the experiments and analyzed the data. YD designed the experiments and edited the manuscript. JZ edited the manuscript. JW and MY designed the experiments and edited the manuscript. All authors contributed to the article and approved the submitted version.

## Conflict of Interest

The authors declare that the research was conducted in the absence of any commercial or financial relationships that could be construed as a potential conflict of interest.

## Footnotes

1 https://www.ncbi.nlm.nih.gov/genome/?term=Anoplophora+glabripennis

2 ftp://ftp.ncbi.nlm.nih.gov/genomes/all/GCF/000/390/285/GCF_000390285.2_Agla_2.0/GCF_000390285.2_Agla_2.0_genomic.gff.gz

## Supplementary Material

Supplementary Figure 1 Comparison of insect experiments. **(A)** *H. cunea* L1 larvae. **(B)** *P. versicolora* L1 larvae. **(C)** *P. versicolora* L2 larvae. **(D)** *P. versicolora* adults. CK, non-transgenic *Populus × euramericana* ‘Neva’; T, transgenic line.

Supplementary Table 1 Information of primers used in the experiment.

Supplementary Table 2 Correlation coefficient.

Supplementary Table 3 Sequencing data evaluation statistics.

Supplementary Table 4 The shared DEGs were identified in two comparative analysis groups.

Supplementary Table 5 The expression information of TFs.

Supplementary Table 6 DEGs identified in the transcriptome of *A. glabripennis* larvae in response to Bt toxin.
