# Supplementary figures and images for: Exogenous Gene Expression and Insect Resistance in Dual Bt Toxin Populus × euramericana ‘Neva’ Transgenic Plants
Source: Front Plant Sci. 2021 May 28;12:660226. doi: 10.3389/fpls.2021.660226 (PMC8193859; doi:10.3389/fpls.2021.660226)

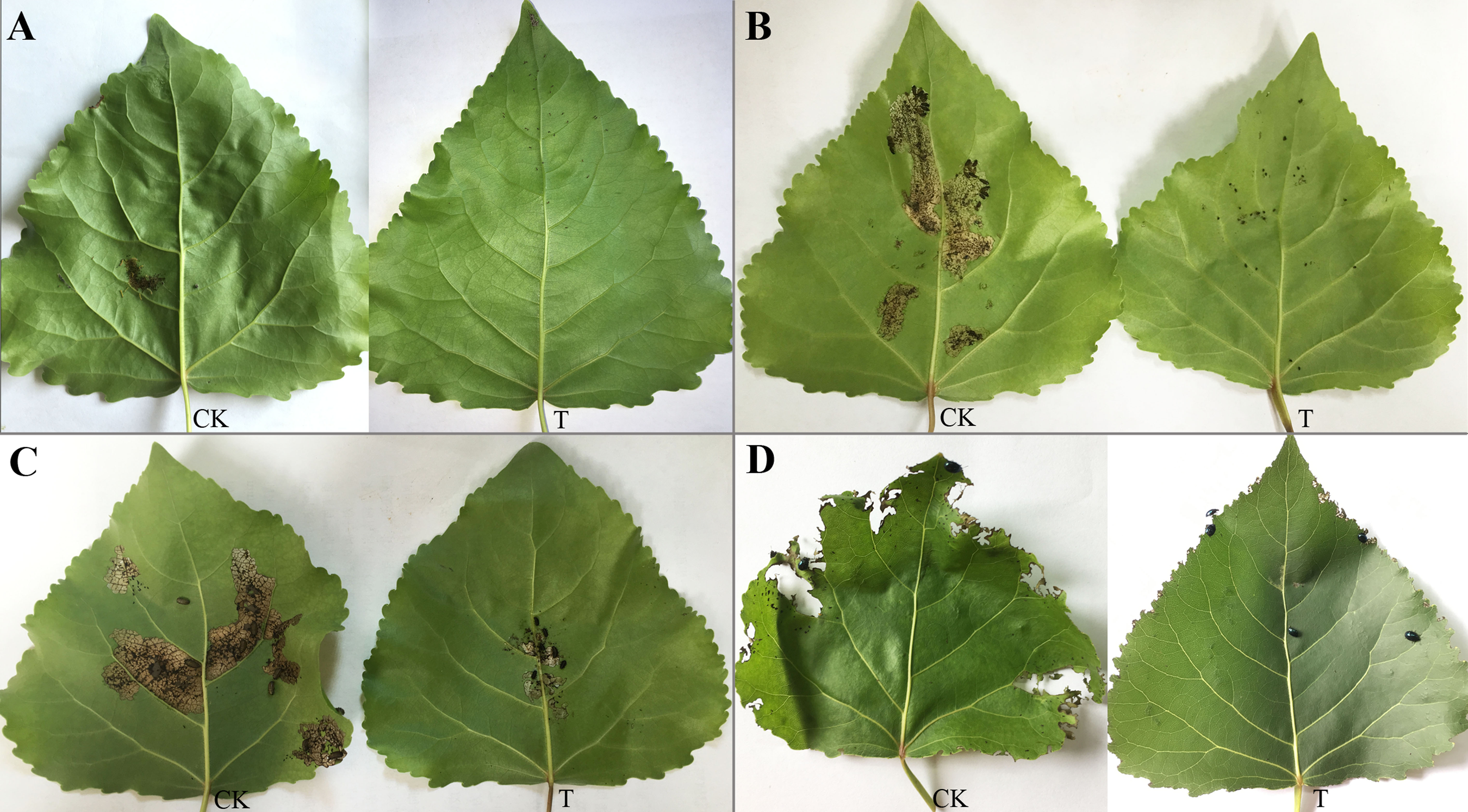

Supplement: Supplementary Figure 1 — Comparison of insect experiments. (A) H. cunea L1 larvae. (B) P. versicolora L1 larvae. (C) P. versicolora L2 larvae. (D) P. versicolora adults. CK, non-transgenic Populus × euramericana ‘Neva’; T, transgenic line. [file Image_1.TIF]
